# A model of integrating convolution and BiGRU dual-channel mechanism for Chinese medical text classifications

**DOI:** 10.1371/journal.pone.0282824

**Published:** 2023-03-16

**Authors:** Xiaoli Li, Yuying Zhang, Jiangyong Jin, Fuqi Sun, Na Li, Shengbin Liang

**Affiliations:** 1 School of Software, Henan University, Kaifeng, China; 2 School of Digital Arts and Communication, Shandong University of Art & Design, Jinan, China; 3 Institute for Data Engineering and Science, University of Saint Joseph, Macao, China; Sejong University, REPUBLIC OF KOREA

## Abstract

Recently, a lot of Chinese patients consult treatment plans through social networking platforms, but the Chinese medical text contains rich information, including a large number of medical nomenclatures and symptom descriptions. How to build an intelligence model to automatically classify the text information consulted by patients and recommend the correct department for patients is very important. In order to address the problem of insufficient feature extraction from Chinese medical text and low accuracy, this paper proposes a dual channel Chinese medical text classification model. The model extracts feature of Chinese medical text at different granularity, comprehensively and accurately obtains effective feature information, and finally recommends departments for patients according to text classification. One channel of the model focuses on medical nomenclatures, symptoms and other words related to hospital departments, gives different weights, calculates corresponding feature vectors with convolution kernels of different sizes, and then obtains local text representation. The other channel uses the BiGRU network and attention mechanism to obtain text representation, highlighting the important information of the whole sentence, that is, global text representation. Finally, the model uses full connection layer to combine the representation vectors of the two channels, and uses Softmax classifier for classification. The experimental results show that the accuracy, recall and F1-score of the model are improved by 10.65%, 8.94% and 11.62% respectively compared with the baseline models in average, which proves that our model has better performance and robustness.

## Introduction

Recently, machine learning and deep learning methods have been widely used in the precision medicine field. For example, El-Sappagh et al. [1] proposed a novel ensemble learning framework for Alzheimer’s disease progression incorporating heterogeneous base learners into an integrated model using the stacking technique, Ali [2] et al. proposed a smart healthcare system for heart disease prediction using ensemble deep learning and feature fusion approaches. F. Ali et al. [3] proposed a new medical monitoring framework based on cloud environment and big data analysis engine to accurately store and analyze medical data and improve classification accuracy. Alfian et al. [4] utilized BLE-based sensor devices and real-time data processing, and uses machine learning algorithms to help diabetic patients better self-manage their chronic diseases. Srinivasu et al. [5] proposed a deep learning-based MobileNet V2 and Long-term Short-Term Memory (LSTM) for classifying skin diseases. Meanwhile, online communities have provided a platform for patients to seek medical treatment and medicine. With the continuous enrichment of medical dialogue text in the social network, analyzing and mining medical text to recommend medical departments for patients is of great significance for improving their experience and diagnosis efficiency.

Medical text is embedded with a large number of medical nomenclature and symptom descriptions. Mining and analyzing medical text can predict diseases and classify patients. Medical text classifications have been applied in various fields of precision medicine, including clinical text classification [6–8], disease features extraction [9, 10], disease features and pathological status [11], phrase detection for medical text [12], extracting biomedical data [13, 14], disease prediction [15, 16], healthcare [17–19], identification of drug interactions [20–23] and identification of adverse drug events [24, 25].

Traditional text classification methods use support vector machine (SVM) [26], Naïve Bayes [27] and other methods to train text word segmentation, statistical word frequency. However, due to the huge dimension of feature vector and the lack of understanding of context, the classification efficiency and accuracy is low. Text classification methods based on deep learning benefit from the powerful capabilities of text feature representation and feature extraction, obviously, these methods are generally better than traditional methods. Yoon Kim [28] proposed TextCNN model, and CNN was first used in the tasks of text classification. On the premise of maintaining the structure of CNN, TextCNN simplifies the number of convolutional layers and input parameters, which effectively improves the training efficiency, but the model has poor interpretability. Liu et al. [29] proposed the TextRNN model, which took the word order and contextual information into account in the feature extraction process. It retained part of the "memory" through the output links and made comparative understanding for long text processing. Lai et al. [30] put forward RCNN model, which combined two-way RNN and maximum pooling operation, made up for the deficiency that one-way RNN only considers the past time information, and paid more attention to the important information of the text through maximum pooling, thus achieving excellent classification results. However, the RNN cycle mechanism is too simple, and it is easy to perform continuous multiplication operations in time when the gradient is backpropagated, resulting in the problems of gradient disappearance and gradient explosion. The improved models such as LSTM and GRU of RNN alleviate the problems of gradient disappearance and gradient explosion to a certain extent. Therefore, LSTM and GRU are used to replace RNN to obtain the contextual information of text [31, 32].

Medical community text often includes topics such as online consultation, fitness and weight loss, intractable diseases, coronavirus disease, and health care. Chinese medical text has a complex structure, contains professional knowledge and has temporal characteristics. Therefore, the efficiency of current Chinese medical text classification methods needs to be improved.

Dogra et al. [33] summarized some machine learning and deep learning algorithms used in the text classification field. Minarro Gimenez et al. [34] applied neural language models to the PubMed corpus, using skip-grams to represent the word representations in PubMed articles. TH et al. [35] used skip-gram and continuous bag of words (CBOW) to test on 1.25 million PubMed articles, verifying the performance of skip-gram and CBOW on large-scale corpora. However, the training speed of the CBOW model is slow. Inspired by the FastText [36] model, some scholars proposed some models such as CNN-LSTM [37] and DC-LSTM [38], and they used FastText to calculate word vectors and used LSTM model to solve the difficulty of information transmission caused by long sequence data. Edara et al. [31] analyzed moods of various cancer affected patients by collecting tweets from different online cancer-supported communities. They used several text mining and machine learning strategies to perform sentiment analysis on distributed LSTM framework and ran it in three different corpora, and the results showed that they were superior to some machine learning methods such as LDA and SVD in accuracy and efficiency. Srinivasu et al. [39] used recurrent neural networks such as LSTM and GRU to predict type 2 diabetes. At the same time, some scholars introduced the attention mechanism [40] into the application fields such as sentiment analysis and text classification, and the results indicate that attention mechanism is of great significance. Attention mechanism extracts meaningful words and sentences, then aggregates these representations to form sentence vectors [41], which is beneficial to the capture of key text features. Zhang et al. [42] proposed a label-based attention for hierarchical multilabel text classification neural network. The model extracted important information from labels at different levels. Lin et al. [43] proposed BertGCN, a model that combined large-scale pretraining and transductive learning for text classification. They used graph to model relationships between different samples from the whole corpus to utilize the similarity between labeled and unlabeled documents, and used GNNs to learn their relationships. However, BertGCN model only used document statistics to build the graph, and it is suboptimal compared with other models. Vulli et al. [44] introduced a novel method for the automated diagnosis and detection of metastases from whole slide images using the Fast AI framework and the 1-cycle policy. Table 1 show the contribution and limitation of some text classification methods.

**Table 1:** Comparison of major works in the field of text classification.

Title	Contribution	Limitation
A Bayesian Classification Approach Using Class-Specific Features for Text Categorization [27]	Propose a Bayesian classification approach for automatic text categorization using class-specific features.	Only four classifications are supported, and the extended classification requires reconstruction of the model, the model has poor scalability.
Convolutional Neural Networks for Sentence Classification [28]	The structure of TextCNN is simple, the training speed is fast, and the classification effect is good, avoiding the process of feature selection in traditional models.	The convolution and pooling operations result in the loss of input information (e.g., lexical order, position), which makes it difficult for the model to learn negation, antonymy and other information in the text sequence.
Recurrent neural network for text classification with multi-task learning [29]	Propose the concept of shared layer, the model supports multi-task learning and pre-training.	Sentence-level corpus classification does not work well.
BertGCN: transductive text classification by combining GNN and BERT [45]	BertCGN combines large scale pre-training and transductive learning for text classification, which have a good performance on wide range of dataset.	Only using documents statistics to build graph structure, it might be sub-optimal.
ZEN: Pre-training Chinese Text Encoder Enhanced by N-gram Representations [46]	Propose a pre-training model for Chinese text, ZEN provides an alternative way of learning larger granular text.	The efficiency of training is low and it needs to be combined with other models for collaborative learning.

Text classification algorithms based on deep learning mainly focus on the design of classifiers, such as local classifiers, global classifiers or their combination classifiers. Especially, in the field of Chinese medical text classification, there are two bottlenecks as follows:

1. Chinese medical text has a wide range of topics, widespread ambiguity, sparse features, and contain temporal features, which leads to low classification efficiency and a sharp drop in accuracy.
2. Chinese medical text contains a large number of medical nomenclatures with complex semantics, and hospital departments have different classification methods, so it is difficult to recommend appropriate departments for patients.

To overcome these limitations which in the current medical text classification approaches, this work mainly studies the information extraction and classification of Chinese medical text using dual-channel mechanism, so that our model takes the temporal and context factors into account, and effectively extracts text features from different granularities. The main contributions of our work are as follows:

1. We use pre-trained word vectors to encapsulate and represent Chinese medical text, so effectively compressing the dimensionality of feature vectors and alleviating data sparsity.
2. We propose a dual-channel Chinese medical text classification model with attention mechanism. The two channels are used to extract text information of different granularities, thereby extracting the temporal and contextual features of Chinese medical text.
3. The experimental results on CMDD and webMedQA datasets show that the proposed model has a great improvement in accuracy, recall, F1-score and balanced accuracy compared with state-of-the-art models.

The rest of this paper is organized as follows: the second section discusses the background of text classification and related research, the third section introduces our methodology and the framework of the proposed model, the fourth section analyzes the experimental results, and the fifth section summarizes our work and provides readers with hints on future directions to extend the proposed models.

## Background and related work

### Word embedding

**Fig 1:**
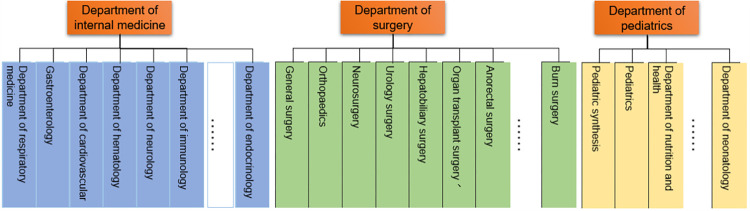
Hospital departments. Different hospitals have different department structures, Fig 1 shows some main departments in a hospital.

**Fig 2:**
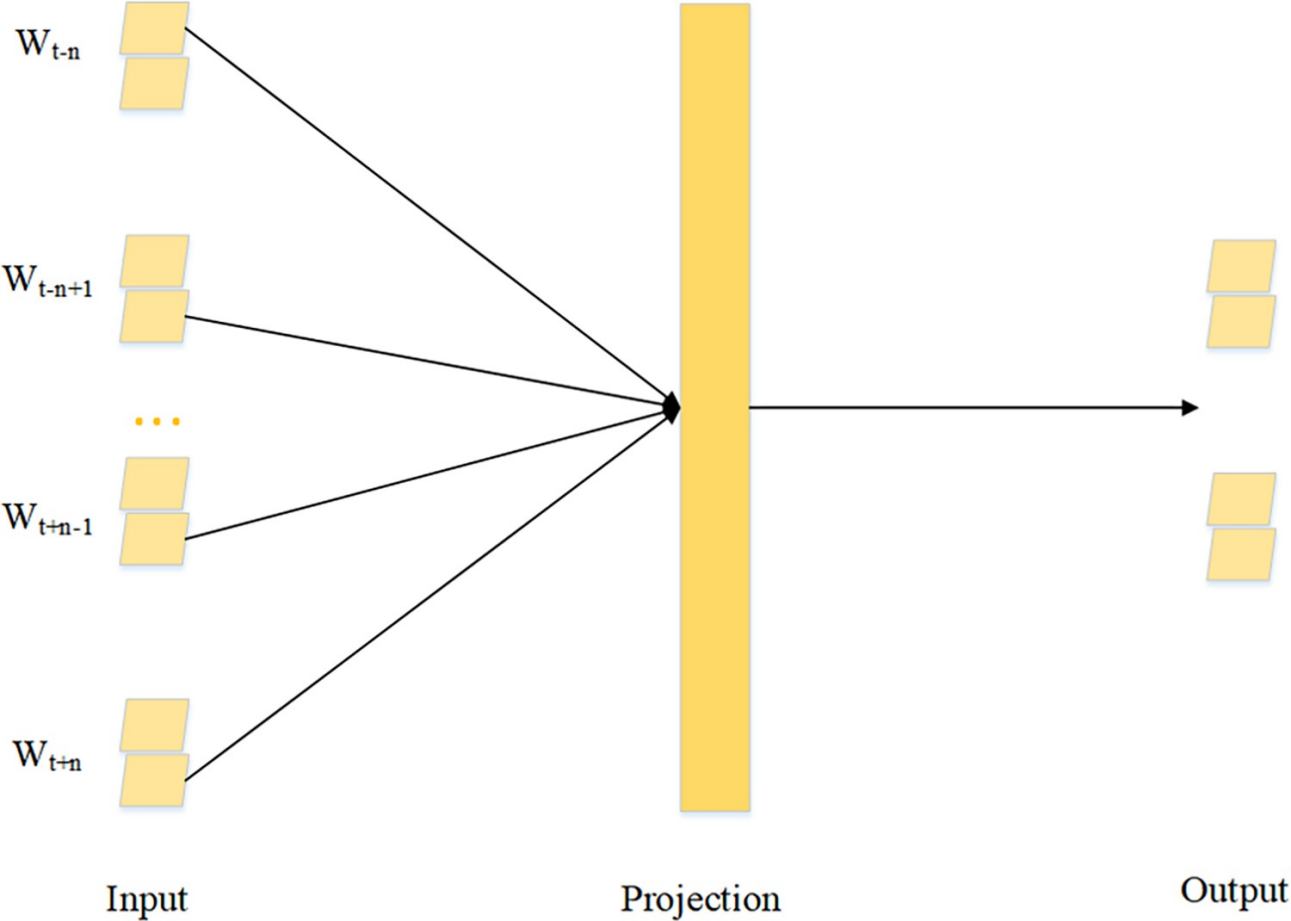
The structure of CBOW. CBOW is a neural network with a 3-layer structure, including input layer, hidden layer and output layer, and performs vector projection operations on the hidden layer. CBOW predicts the central word through the context.

The traditional vector space model (VSM) leads to a high dimension vector, and the features are independent of each other, which is inconsistent with the requirement that long text needs to be associated with the context. Bengio [47] proposed neural network language model (NNLM) and put forward the distributed representation of words in text for the first time. The distributed representation of words, that is, word vectors, can make the spatial distance between semantically similar words closer, thus containing more semantic information. The lower dimensions made it possible to train larger corpus and easier to solve the problem of dimensional disaster. Later, some tools such as Word2Vec [48], GloVe [49], FastText [36] for training word vectors appeared. This work uses the Word2Vec model to construct word vectors. Word2Vec includes two diametrically opposed models, skip-gram, and CBOW. The CBOW model trains the contextual word vector and outputs the word vector of the specific words. Usually, the department classification of a hospital is shown in Fig 1, and we utilize the CBOW method to encode the name of the department. The structure of the CBOW is shown in Fig 2.

### GRU

**Fig 3:**
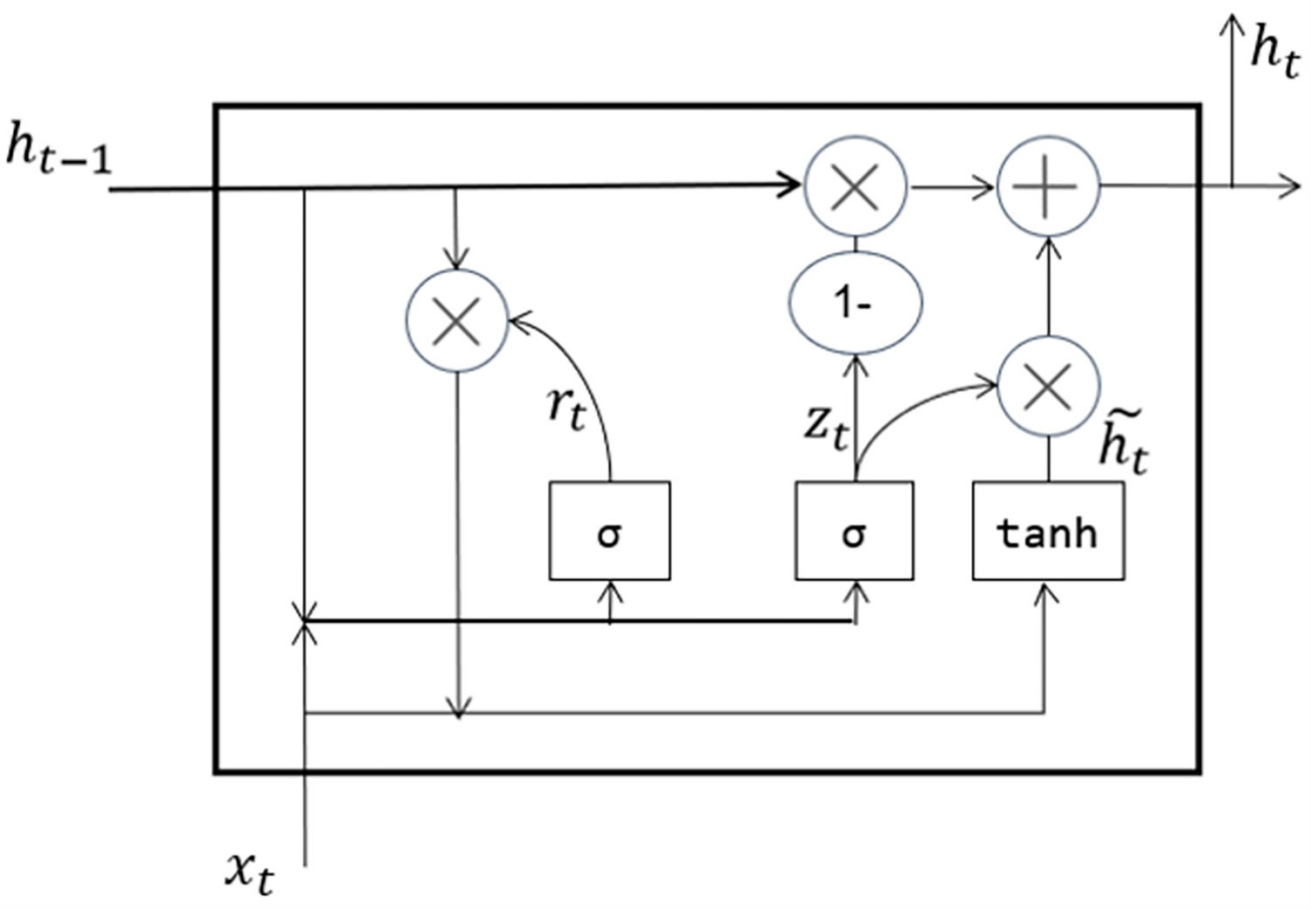
The structure of GRU. GRU only contains update gate and reset gate, and the structure is simpler. The update gate is used to control the degree to which the state information of the previous moment is brought into the current state. The larger the value of the update gate, the state information of the previous moment is brought into more. The reset gate controls how much information from the previous state is written into the current candidate set ${\tilde{h}}_{t}$.

The GRU [50] is a kind of Recurrent Neural Network (RNN), the GRU structure is a gated network structure. GRU solves the problem of gradient disappearance in long-term memory and backpropagation. Compared with LSTM, its structure is simpler, it only has two gates: update gate and reset gate. Therefore, less parameters need to be trained and calculated, and the training efficiency is higher. It is widely used in natural language processing tasks. The GRU structure is shown in Fig 3.

The GRU model uses Eqs 1–4 to update the parameters.


$$Z_{t}=\sigma (w_{z}x_{t}+U_{z}h_{t-1}),$$ (1)

$$r_{t}=\sigma (w_{t}x_{t}+U_{t}h_{t-1}),$$ (2)

$${\tilde{h}}_{t}=\tanh(wx_{t}+U(r_{t}\circ h_{t-1}),$$ (3)

$$h_{t}=(1-z_{t})\circ h_{t-1}+z_{t}\circ {\tilde{h}}_{t},$$ (4)

where *z*_*t*_ is the update gate of the GRU, *r*_*t*_ is the reset gate of GRU. The update gate controls the degree which the state information of the previous moment is transmitted to the current moment, and the reset gate controls the degree which the state information of the previous moment is forgotten. *w*_*z*_, *w*_*t*_ and *w* are weights of GRU. GRU obtains two gated states through the hidden state information *h*_*t*−1_ at the previous moment and the node inputs *x*_*t*_ at the current moment. After obtaining the gated state, the reset gate obtains forgotten state, then fuses *x*_*t*_ at the current moment and activates it through the nonlinear function tanh, finally the update gate selects and memorizes the input of the current node.

The GRU is calculated from the front to the back according to the natural language order, only considering the impact of the above words in the text and the overall text semantics, ignoring the impact of the following words on the above and the overall semantics. Therefore, the bidirectional GRU (BiGRU) simultaneously propagates the calculation processing for the text input in positive order and reverse order, and integrates the corresponding position output, so that the text semantic information is more comprehensive and accurate.

### Attention mechanism

**Fig 4:**
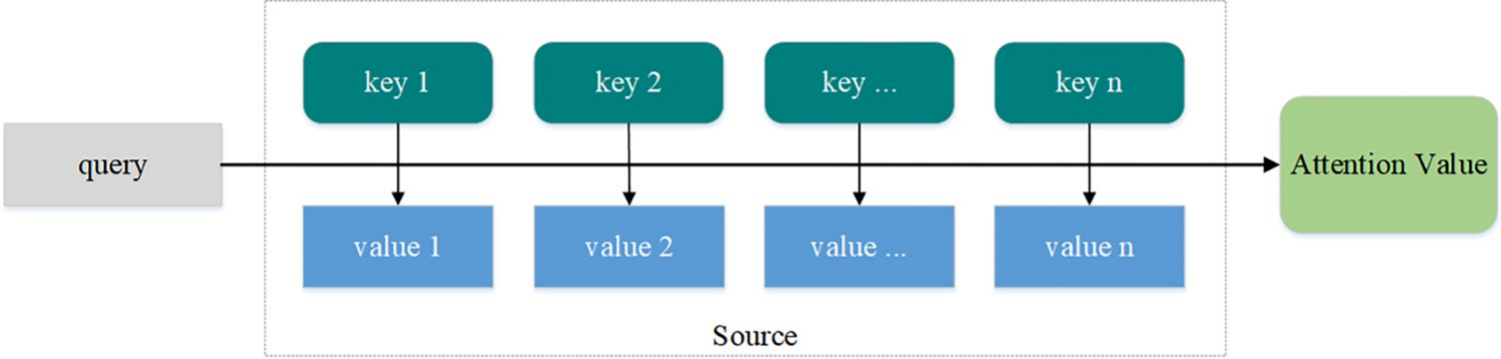
Attention mechanism. Attention is a set of attention distribution coefficients, and the attention value is obtained by using the attention function. The elements in Source are imagined as a series of <key, value> pairs. At this time, specify a query in the target, and calculate the similarity or correlation between query and each element to obtain the weight coefficient of each key corresponding to value, and then the value is weighted and summed to get the final attention value.

The attention mechanism [51] was first proposed by the Google Mind team and applied to the field of image processing. It can be used by the multilayer structure of the network model for a single feature of an input sequence. However, in some scenarios, different models focus on the input sequence differently, that is, multiple features represent different aspects of the input sequence. The attention mechanism assigns different weight to these different representations. The more important the information, the higher the weight assigned, then greater influence on the classification. On the contrary, other information representations can be ignored to reduce the noise and redundancy of the input sequence to achieve better classification. The calculation of the attention mechanism can be divided into two steps: the first step is to calculate the attention distribution in all input sequences, and the second step is to calculate the weight of the input information according to the attention distribution. The attention mechanism can essentially be described as a map of a query to a series of key-value pairs, as shown in Fig 4.

## Methodology

**Fig 5:**
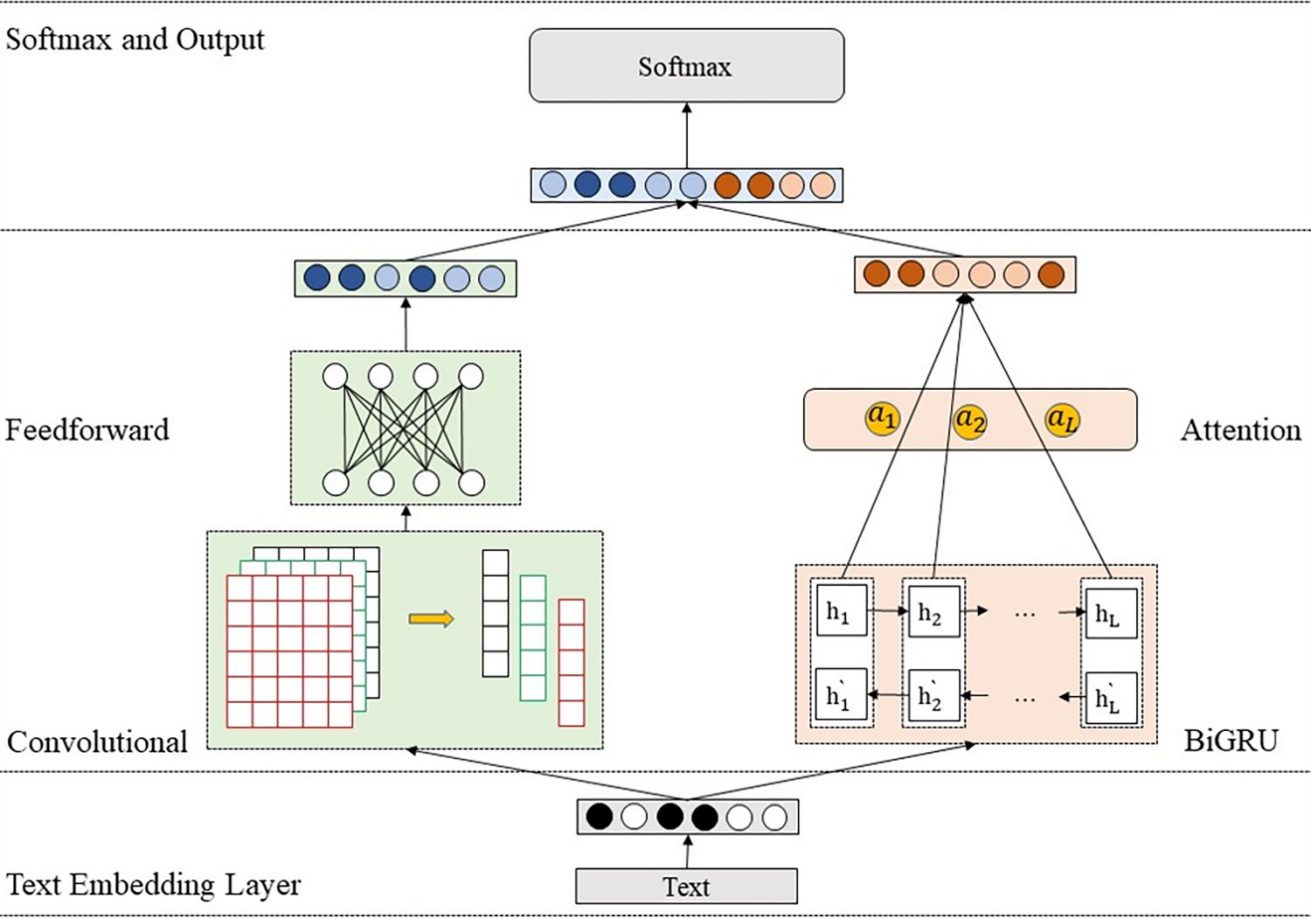
The framework of Dual-CGA. The model includes dual channels, four-layer structure. The dual channels are located in the convolutional and feedforward neural network channel layer. This layer is the core of the entire architecture, and integrated dual channels process text features of different granularities.

The Dual-channel CNN-BiGRU-Attention (Dual-CGA) model proposed in this paper is a hierarchical hybrid text representation model. The overall structure is as shown in Fig 5. It is mainly composed of several parts: input embedding layer, convolutional and feedforward neural network channel layer, BiGRU and attention channel layer, and Softmax classification output layer. The model adopts a dual-channel mechanism. One channel uses convolution operations to obtain local information of text with different granularities, and the other channel uses BiGRU and attention mechanism to obtain global contextual semantic information. Finally, a full connection layer and Softmax classification are used to determine the classification of the outpatient department.

### Medical text embedding layer

The medical text embedding layer aims to map the input text into a vector matrix composed of word vectors. The model uses the CBOW mode of Word2Vec to obtain the word vector as the representation element of the text vector. CBOW model predicts the probability of the target words through the window context and finally updates the parameter matrix, that is, the trained word vector representation. The word vector obtained by Word2Vec is generally from 100 to 300 dimensions, which is smaller than the one-hot coding dimension, so as to effectively avoid dimension disaster and data sparsity. Moreover, the word vector obtained by text training can better represent the semantic between words.

In this paper, word vectors are used in two ways: loading pre-trained word vectors and randomly initializing word vectors. The first step of both methods is to construct the vocabulary corresponding to the training set and then map the word sequence into a digital index sequence. By loading the pre-trained word vectors, we traverse and extract the word vector matrix corresponding to the vocabulary and generate word vector matrix by the text numerical index during the training stage. The strategy of randomly initializing word vectors is to convert text-digit sequences into randomly initialized word vectors trained with the model in the training stage, without loading pre-trained word vectors. The generation of the input text vector representation matrix *W* has the following steps:

**Step 1:** Segment each piece of data in the training set and construct a vocabulary list.

**Step 2:** For each piece of text in the training set and the test set, map the word segmentation result to the corresponding digital index of the vocabulary in which the index of words that have not appeared in the pre-trained vocabulary are filled with -1, and the word vector corresponding to the -1 index uses random initialization processing.

**Step 3:** Data length normalization operation, according to the data statistic result, the length of normalized value is set to 100, the excess part is truncated, the data with insufficient length is filled with 0, and the word vector corresponding to 0 index is initialized with all 0.

**Step 4:** Load the pre-trained word vectors, the index corresponding to the data is converted into word vector to obtain the word vector matrix *W*.

Through the above steps, the word vector matrix representation *W* is finally obtained, and *W* is used as the input of the model.

### Convolutional and feedforward neural network channel layer

After the processing of the text embedding layer, the matrix representation of the word vector is obtained, but the word vector depends on the effect of word segmentation. Especially in Chinese medical text, there are many medical nomenclatures and terms. Poor word segmentation brings ambiguity and errors in text presentation into text encoding, resulting in greater errors in the next task. In addition, adjacent words in Chinese text are related, and the combination of words forms a structure containing more important contextual semantic information, such as subject-predicate, verb-object. The overall meaning of this structure can better represent the text. The Dual-CGA model uses convolution kernels to perform convolution operations on text according to different length of text, similar to n-grams, the model learns the semantic information contained in text of different granularities and covers multiple words of different length, thereby improves text semantics representational capabilities and performance on downstream text classification tasks.

**Fig 6:**
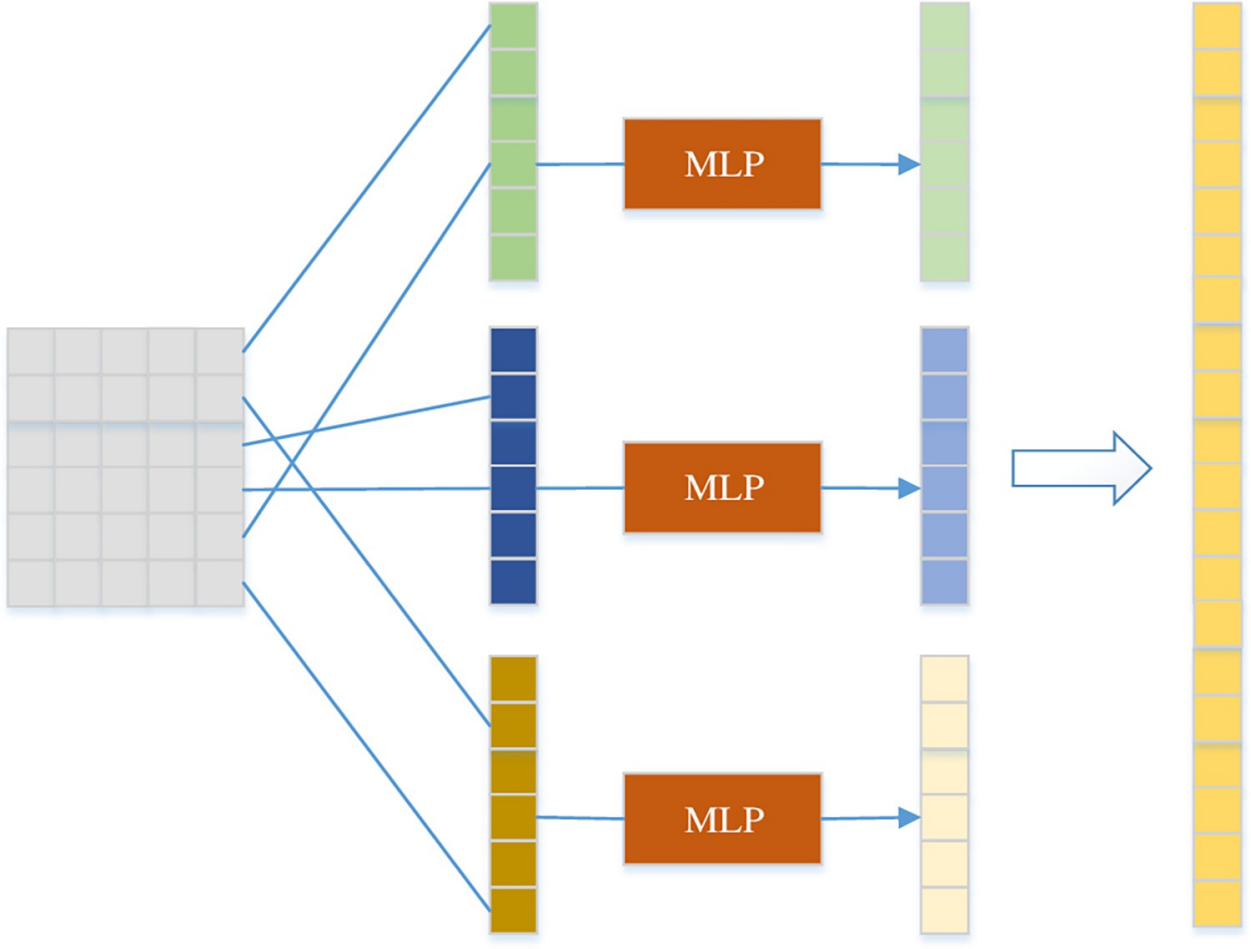
The structure of convolution and feedforward neural network channel layer. This layer firstly computes feature vectors by a convolutional structure consisting of three filters of different sizes, and then global information is obtained by MLP.

The dimension of the word vector in this layer is 300 dimensions, and the convolutional neural network uses three filters, the sizes of which are 3*300, 4*300, and 5*300. Usually, each filter contains several feature maps. The filter only outputs a feature map vector, and then each feature map passes through the MLP layer. The MLP layer is equivalent to a matrix mapping with a global receptive field. It assigns weights to the results of convolution operations with different granularity and dynamically adjusts the parameters of the mapping matrix with network training, finally achieving the purpose of allocating high weight to important granularity information. The channel layer structure is as shown in Fig 6. This residual connection and hierarchical normalization operation can well prevent overfitting and reduce the occurrence of gradient disappearance during backpropagation, thus making Dual-CGA more robust.

The calculation process of this layer is as shown in Eqs 5–9,

$$c_{3},c_{4},c_{5}=Conv(x),$$ (5)

$$c_{3}^{'}=f(w_{3}\cdot c_{3}+b_{3}),$$ (6)

$$c_{4}^{'}=f(w_{4}\cdot c_{4}+b_{4}),$$ (7)

$$c_{5}^{'}=f(w_{5}\cdot c_{5}+b_{5}),$$ (8)

$$c=[c_{3}^{'},c_{4}^{'},c_{5}^{'}].$$ (9)

Among them, Eq 5 is convolution operation, which generates three feature vectors: *c*_3_, *c*_4_, *c*_5_. Then input the feature vectors into the MLP network respectively and obtain the hidden layer output $c_{3}^{'},c_{4}^{'},c_{5}^{'}$, where *w*_3_, *w*_4_, *w*_5_ and *b*_3_, *b*_4_, *b*_5_ are the parameter matrix of the MLP network layer and bias vector. *c* is the concatenated long vector, that is, the text feature representation of the convolutional and feedforward neural network channel layer.

### BiGRU and attention channel layer

**Fig 7:**
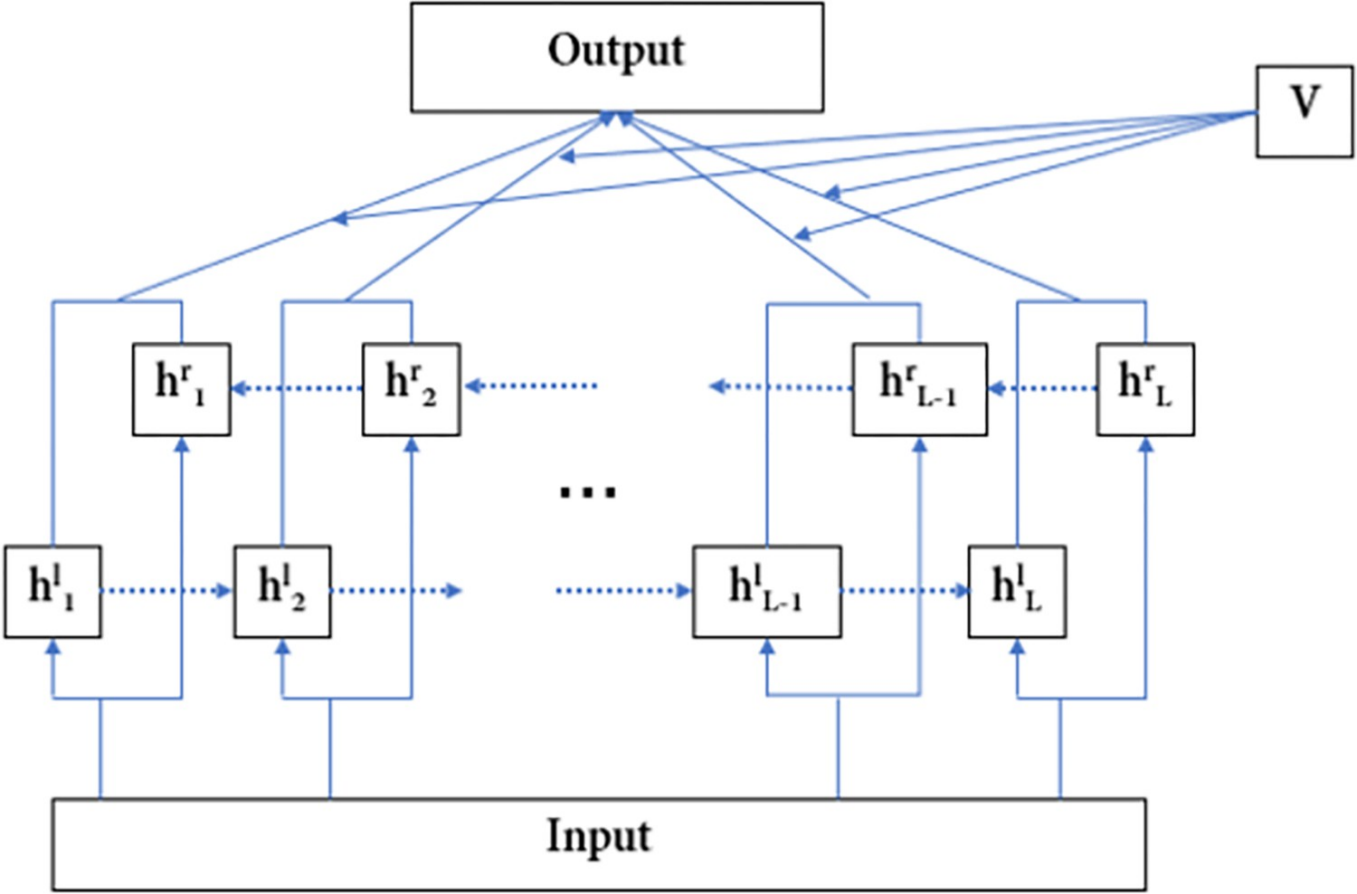
The structure of BiGRU and attention layer. This layer is composed of BiGRU and attention mechanism, which is used to fully obtain the long text context, and evaluate the importance of BiGRU output at each moment through the attention mechanism.

In order to obtain more comprehensive text information, the model uses the BiGRU network to extract text information. The BiGRU network can extract the sequence features of the text sequence in the forward and reverse directions respectively. At the same time, the influence and relationship of context are considered; then, the text feature representation vector of the channel is obtained. The structure of BiGRU and attention channel layer is as shown in Fig 7.

$h^{l}{}_{t}=LSTM^{l}(x_{t},h_{t-1})$ is the feature output of the forward hidden layer and $h^{r}{}_{t}=LSTM^{r}(x_{t},h_{t-1})$ is the output of the reverse hidden layer. The hidden layer features in both directions are spliced together, that is, $h_{t}=[h^{l}{{}_{t}}_{'}h^{r}{}_{t}]$, to get the output of this layer.

For the RNNs, the final output of the forward and reverse GRU network is spliced together and directly used as the text representation as the input of the text classifier. This method is relatively simple. Although the LSTM and GRU networks can process longer sequences, they can also learn longer text contextual features, there is still information attenuation. For classification tasks, this feature attenuation is fatal, resulting in low classification effects. In order to address this problem, the literature [34] proposed another way: splicing the output of the hidden layer at each moment together and then using the maximum pooling to obtain the most important feature representation as the input of the text classifier, which highlighted the most important features in the text. This method highlighted the most important features in the text, but ignored other secondary important features. In order to take into account the characteristics of these different importance levels, our model introduces the attention mechanism to measure the importance of the output of GRU network at each time, so as to calculate the weight of the output at each time. The calculation method is as shown in following equations:

$$s(x_{i},q)=x_{i}^{T}q$$

$$a_{i}=softmax(s(x_{i},q))$$ (10)

$$V_{a}=attention(V)=\sum_{i=1}^{N}a_{i}V_{i},$$

$$e_{t}=u_{a}^{T}\tanh(V_{a}\cdot h_{t}+b_{a}),$$ (11)

$$a_{t}=\frac{\exp(e_{t})}{\sum_{i=1}^{L}\exp(e_{i})}.$$ (12)

Eq 10 represents the computing method of the attention mechanism, Eq 11, *e*_*t*_ stands for *h*_*t*_ corresponding hidden layer output, *V*_*a*_ is the attention weight matrix, $u_{a}^{T}$ is the random initialization vector, *b*_*a*_ is the bias. *a*_*t*_ in Eq 12 represents the normalized output of each hidden layer *e*_*t*_, that is, the weight of the state output *h*_*t*_ at time *t*. After calculating the weight, the final representation can be obtained, as shown in Eq 13,

$$v_{t}=\sum_{t=1}^{L}a_{t}\cdot h_{t},$$ (13)

The text feature representation of the final Bi-GRU and attention channel layer is recorded as *v*_*t*_.

### Softmax and classification output layer

The text feature representationr isis fused and spliced into a long vector to obtain the final text feature representation, denoted as *o*, as shown in the following equation:

o=c⊕v, (14)

The final text feature representation is input into the Softmax classifier, and gethe results is the probability distribution of the categories. The classification with the largest probability is selected as the final classification result.

## Experiments

In order to verify the reliability of Dual-CGA model, the complete contrast experiments are designed. The two datasets of the Chinese medical dialogue data (CMDD) and webMedQA are used to compare the Dual-CGA model with other baseline models.

### Datasets and hyperparameters

**Fig 8:**
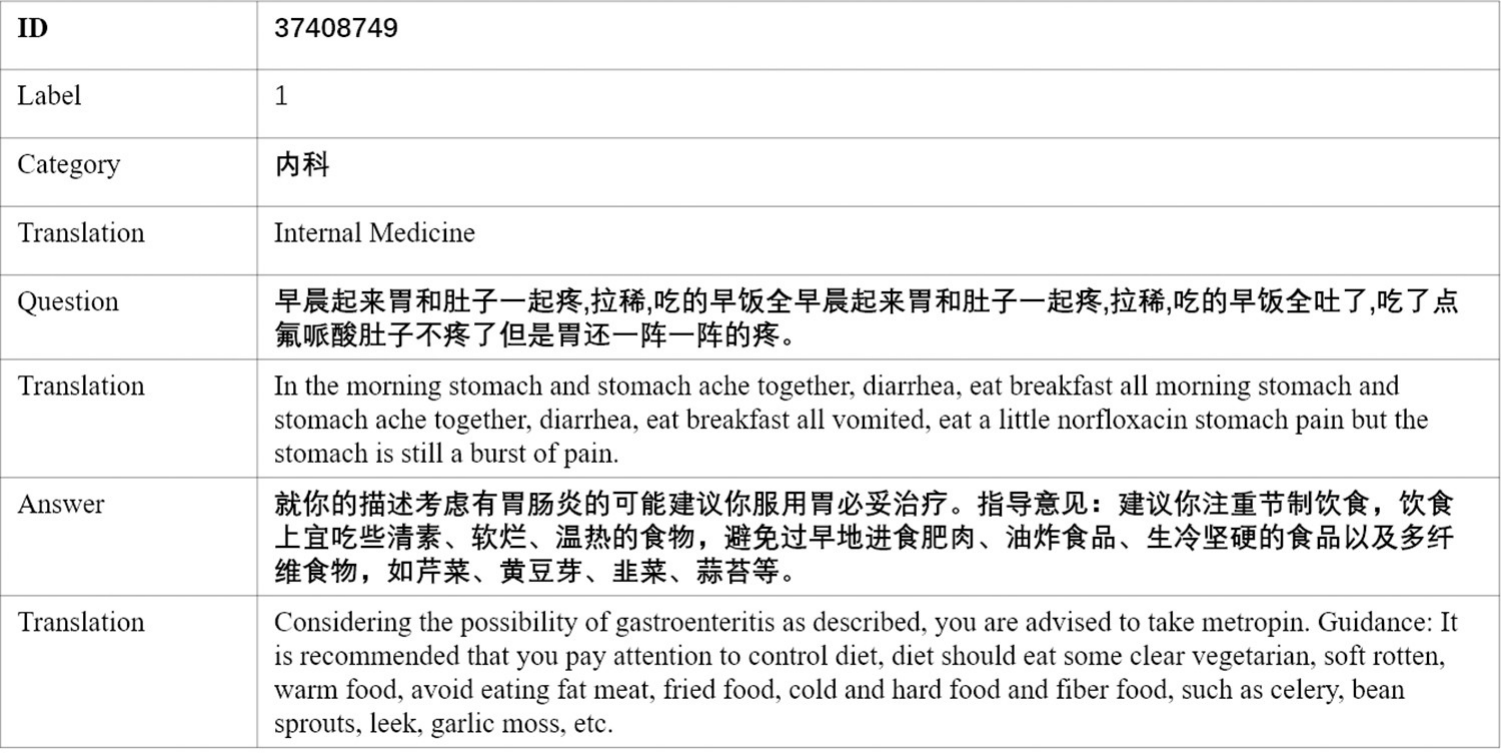
A record sample of webMedQA. A record consists of 5 fields: ID, label, category, question and answer.

We conduct experiments on two Chinese medical public datasets of different scales, among which the larger dataset is CMDD, which is a public medical question answering dataset. The dataset contains 6 folders corresponding to 750,000 question-answer pairs related to 6 different outpatient departments. Specifically, the number of detailed records corresponding to each department is as follows: andriatria (94,596 records), IM (220,606 records), OAGD (183,751 records), Oncology (75,553 records), Pediatric (101,602 records) and Surgical (115,991 records). Another dataset is the webMedQA, which is a question and answer dataset about Chinese medical questions collected from online healthcare websites. It contains more than 50,610 questions in 23 different categories,Each question has 1 positive and 4 negative answers. A sample is shown in Fig 8.

After data processing, The statistical results of the available data are described in Table 2.

**Table 2:** Statistics of datasets.

Dataset	Categories	Records	Average length of records
CMDD	6	752,236	389.43
webMedQA	23	50,610	86.68

As for how to define the confusion matrix under multi-classification, we take the CMDD dataset as an example, and define the labels corresponding to the 6 categories of the dataset as 0–5 in turn, and the TP, TN, FP and FN with category 0 are calculated as follows:

TP: the actual value is 0, and the predicted value is also 0;

FP: the predicted value is 0, the actual value is not 0;

FN: the actual value is 0, the predicted value is not 0;

TN: neither the actual value nor the predicted value is 0.

It is well known that the quality of hyperparameters will directly affect the training effect of the model, so it is important to choose a series of optimal hyperparameters. The settings of the hyperparameters are shown in Table 3.

**Table 3:** Hyperparameters.

Parameters	Values
Vector dimensions	300
Batch size	128
Kernel size	3/4/5
Learning rate	1e-3
Dropout	0.25
Optimizer	Adam

This experimental environment is based on 64 bit Anaconda as the modeling platform, and the operating system is Ubuntu 20. The experimental environment is shown in Table 4.

**Table 4:** Experimental environment.

Hardware and software	Values
CPU	Intel i9 10900K
RAM	128G
GPU	GeForce RTX 3090
Display RAM	24G
OS	Ubuntu 20
Development language	Python 3.9
Python distribution platform	Anaconda 64-Bit
ML Platform	TensorFlow 2.2
Chinese word segmentation tool	Jieba 0.42.1
Scientific computing package	NumPy 1.19.5

### Baselines and evaluation

This paper conducts experiments on two datasets with 1 experimental group and 8 control groups. The experimental group was designed based on the Dual-CGA model, while the control groups used 3 basic text classification models, Naïve Bayes [27], SVM [26] and CNN [28], as well as RCNN [30], BiGRU [50], BiLSTM, Transformer [45], ZEN [46] and InducT-GCN [52] models.

- Naïve Bayes: Naïve Bayes assumes the conditional independence of conditional probability distribution, that is, ignores the relationship between features, making each feature a separate hypothesis. When using Naïve Bayes for text classification, it is more effective in the case of less data, but it is sensitive to input data, especially Chinese medical text.
- SVM: a benchmarked supervised-learning approach that can handle nonlinear classification. The aggregation words proposed by VSM are embedded as features.
- CNN: a classic variant of convertional deep neural networks that implements convolutional filters with learned weights and bias.
- BiGRU: BiGRU can calculate the input sequence from front to back, which extract the features more accurately and grasp the context relationship of medical text.
- BiLSTM: a bidirectional LSTM developed by [53] with a concatenated layer of one forward LSTM and one backward LSTM and a hidden state.
- Transformer: Transformer can train all words in parallel at the same time, which greatly speeds up the computational efficiency. Moreover, Transformer adds location embedding to help the model understand the order of language. The structure of Transformer includes self-attention and full connection layer.
- ZEN: The model [46] is a BERT-based Chinese text encoder enhanced by N-gram representations that take into account different combinations of characters during training.
- InducT-GCN: The model [52] is a inductive graph-based text classfication framework without using extra resources.

We use some popular evaluation indicators in machine learning to evaluate the performance of the models, including accuracy, recall, F1-score and balanced accuracy (BA), as shown in Table 5.

**Table 5:** Evaluation indicators.

Name	Description
Accuracy	$Accuracy=\frac{TP+TN}{TP+FP+TN+FN}$
Recall (R)	$R=\frac{TP}{TP+FN}$
Precision (P)	$P=\frac{TP}{TP+FP}$
F1-score	$F1-score=\frac{2*P*R}{P+R}$
Balanced Accuracy (BA)	$BA=\frac{1}{2}(\frac{TP}{TP+FN}+\frac{TN}{TN+FP})$

Accuracy represents the overall prediction ability of the deep learning model. True positive (TP) and true negative (TN) measure the ability of classifier models to predict the correct department to recommend for the patients. False positive (FP) and false negative (FN) identify the number of incorrectly predictions generated by the models. Recall measures sensitivity of the classifier models. F1-score keeps the balance between precision and recall. It’s often used when class distribution is uneven, but it can also be defined as a statistical measure of the accuracy of an individual test. Balanced accuracy is useful for multiclass classification, BA is the average of recall obtained in each class.

### Results and analysis

In order to avoid model overfitting as much as possible, the optimization method we choose is the dropout method. The mechanism of this method is to delete some nodes during propagation, so that we can reduce the network complexity, and the established neural network model will not become overfitting. The experimental results are shown in Tables 6 and 7.

**Table 6:** Experimental results on CMDD.

Models	Accuracy (%)	Recall (%)	F1-score (%)	BA (%)
NB	82.78	82	82	83.92
SVM	81.63	79	81	82.96
CNN	87.33	86.33	86.76	87.96
RCNN	88.46	87.9	88.02	88.98
BiLSTM	88.49	87.92	87.99	89.02
BiGRU	88.85	88.17	88.38	89.35
Transfomer	88.15	87.94	87.66	88.72
ZEN	90.65	90.31	90.68	91.06
InducT-GCN	82.03	82.04	82.03	83.4
**Dual-CGA**	**90.8**	**90.53**	**90.51**	**91.16**

**Table 7:** Experimental results on webMedQA.

Models	Accuracy (%)	Recall (%)	F1-score (%)
NB	67.32	37	37
SVM	64.2	36	37
CNN	73.13	33.95	34.81
RCNN	68.25	30.93	32.74
BiLSTM	69.91	34.6	33.5
BiGRU	71.39	36.72	36.19
Transfomer	71.69	37.3	39.2
ZEN	23.45	16.66	6.33
InducT-GCN	61.72	61.72	61.72
**Dual-CGA**	**75.11**	**42.2**	**45.06**

**Fig 9:**
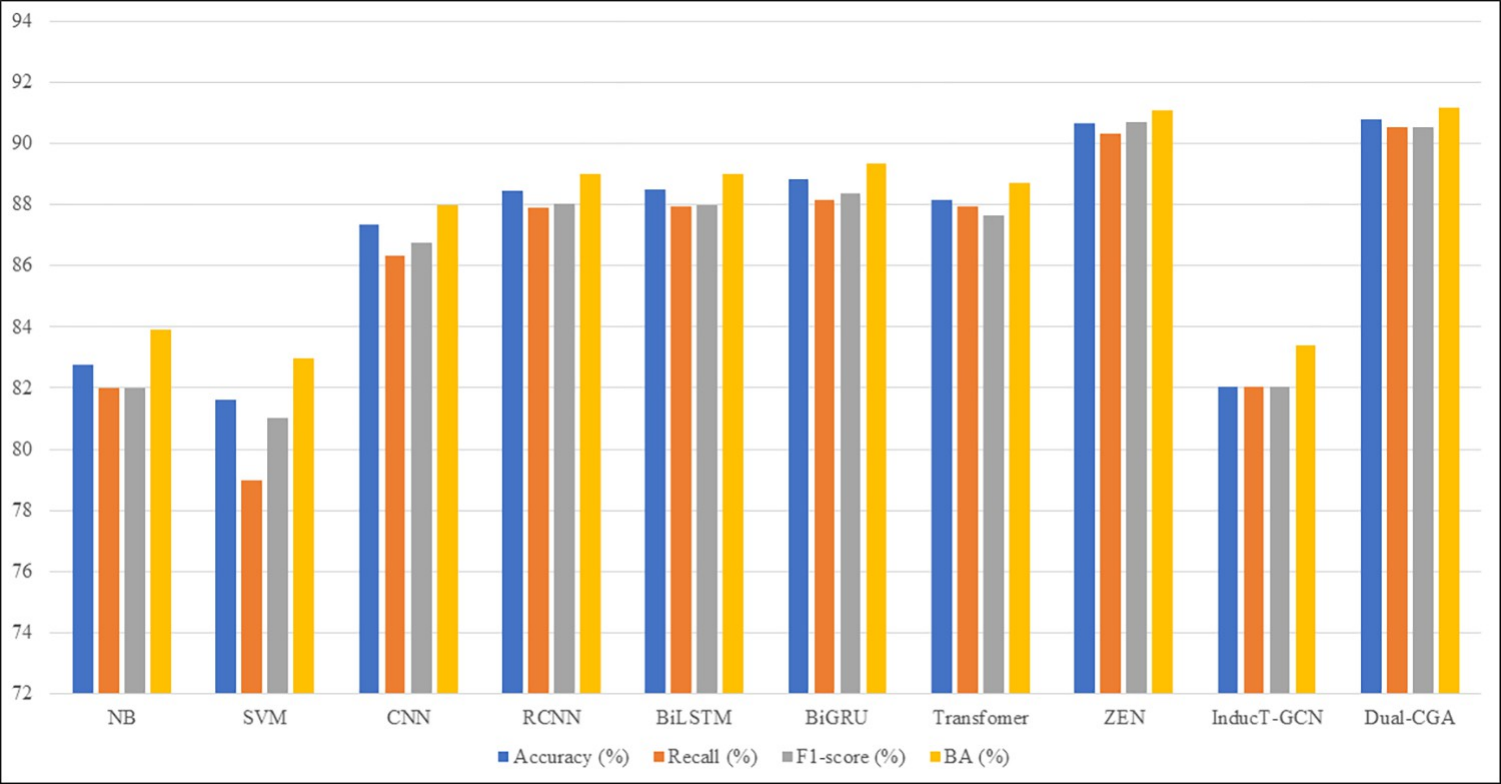
Experimental results on CMDD dataset. The experimental results in the CMDD dataset show that Dual-CGA is almost superior to the baseline models in the four indicators of accuracy, recall, F1-score and BA.

**Fig 10:**
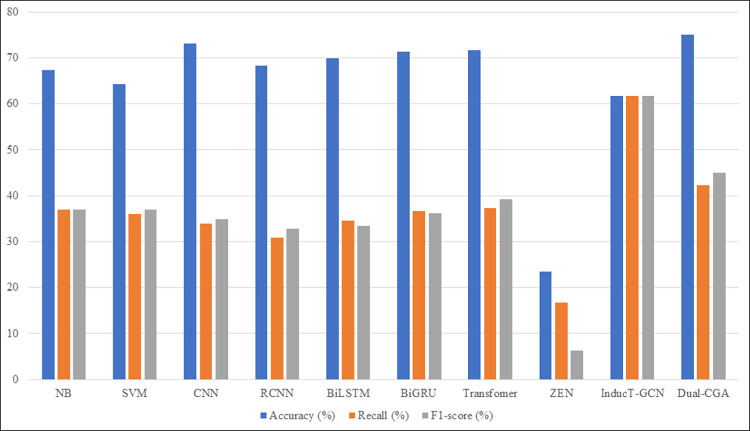
Experimental results on webMedQA dataset. The experimental results on the webMedQA dataset show that Dual-CGA also has certain advantages in indicators such as accuracy, recall, and F1-score, but it is inferior to inductT-GCN in terms of recall and F1-score, because the average text length of the dataset is shorter, the ability of BiGRU to extract short text features is not as good as that of the pre-trained structure model.

It can be seen from Tables 6 and 7 that the Dual-CGA model proposed in this paper is superior to other models such as CNN, RCNN, BiLSTM, BiGRU, Transformer and InducT-GCN in terms of accuracy, recall, F1-score on CMDD dataset but slightly lower than ZEN model in terms of F1-score. However Dual-CGA shows an advantage on the webMedQA dataset, which is much better than other comparison models in terms of accuracy, recall and F1-score. Figs 9 and 10 describe the comparison between the Dual-CGA model and other baseline models. It is observed that the performance of our model is better than other models.

Compare Figs 9 and 10, it can be seen that the results of the Dual-CGA model are quite different. In fact, not only the Dual-CGA, but also other models, the main reason lies in the dataset itself. For example, the text average length of webMedQA is only 86.68 characters, and its average length is only one-fourth of the CMDD dataset, resulting in the number of extracted features is small. In addition, the webMedQA dataset has only more than 40000 records, but the amount of its catalogs is 1.83 times great than that of CMDD dataset, resulting worse experimental results on webMedQA. The InducT-GCN model is a corpus-level text classification model, which has certain advantages in short text classification. This also points out the direction for our future research, that is, to improve classification effect of short text corpus. Moreever, Dual-CGA has better performance than baseline models, among them, it is the model with the shortest traning time.

### Ablation studies

We conduction ablation studies on CMDD dataset, we successively adjusted three different learning rates, then removed the attention mechanism, and adjusted the kernel size. In these different hyperparameter states, there is a significant impact on the execution results of the model, as shown in Table 8.

**Table 8:** Ablation tests on different hyperparameters.

Hyperparameters	Accuracy	Recall	F1-score
Without attention	0.8985	0.8914	0.8931
Kernel size (2/3/4)	0.9003	0.8904	0.8937
lr = 1e-4	0.8950	0.8868	0.8891
lr = 1e-2	0.8756	0.8651	0.8693
lr = 1e-5	0.8585	0.8466	0.8495

**Fig 11:**
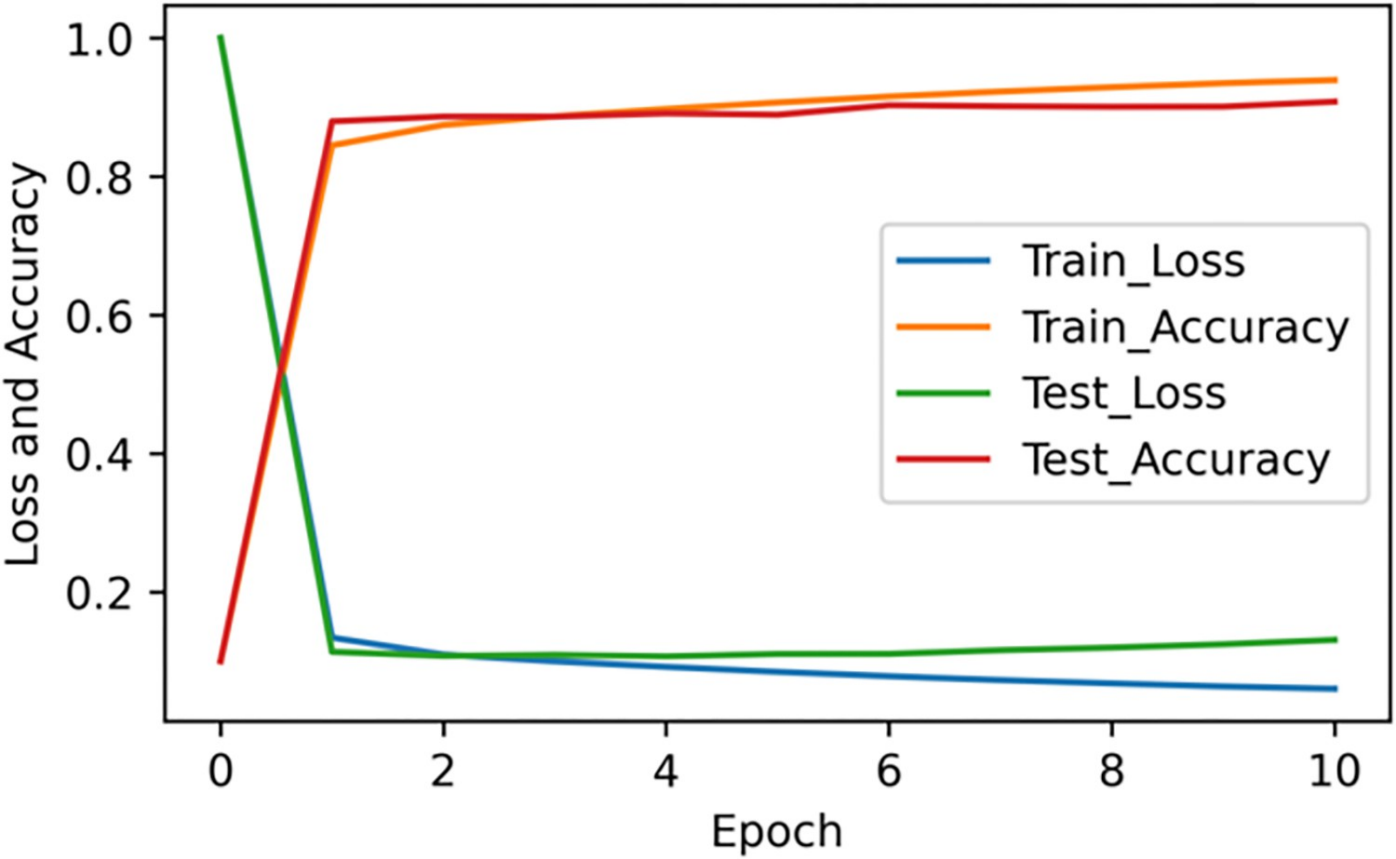
Loss associated with batches processed before fine-tuning the model. The performance of the Dual-CGA model in the training phase is consistent with that in the test phase.

Fig 11 shows that the performance of the model’s accuracy and loss values during the training phase is basically consistent with the performance during the test phase, and the model does not have the problem of overfitting.

**Fig 12:**
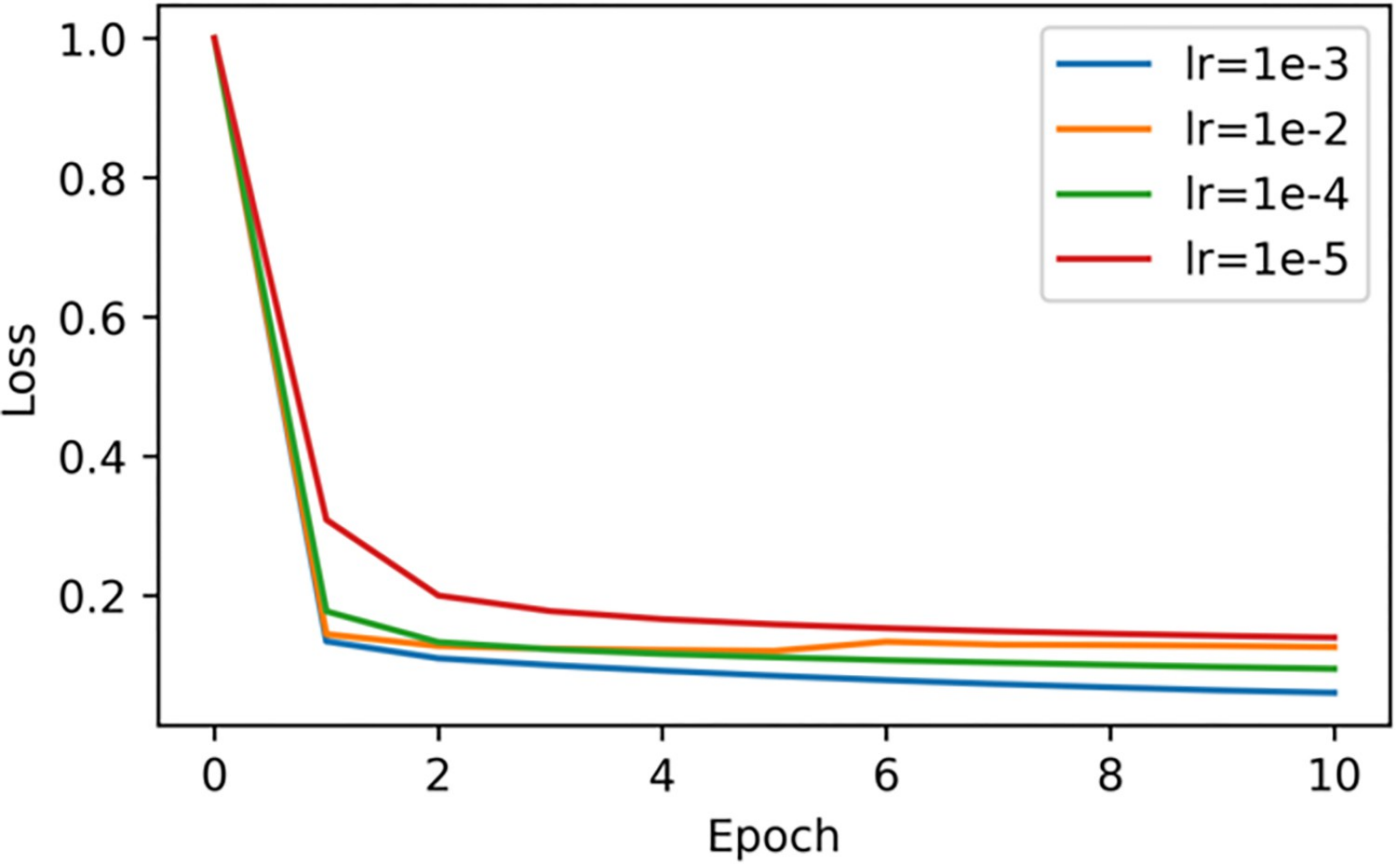
The loss value with different learning rate. Fig 12 shows that when the learning rate is 1e-3, the loss value is the smallest.

We adjust different learning rates and compare the performance of the loss value under the four different values of the learning rate of 1e-2, 1e-3, 1e-4 and 1e-5. Fig 12 clearly shows that when the learning rate is 1e-3, the loss value is lowest.

## Conclusions

This paper solves a problem of accurately recommending medical departments for patients based on their symptom descriptions. We focus on the structural features of Chinese medical text and propose a Chinese medical text classification model based on multi-channel and attention mechanism, namely Dual-CGA. The model adopts a dual-channel method including convolutional neural network and BiGRU, which is used to identify different granularities and medical nomenclature of Chinese medical care. At the same time, an attention mechanism is introduced to increase the weight of important word vectors, and have a good classification effect. A large number of experimental results show that our model has good performance in text classification, the accuracy, recall and F1-score are respectively 10.65%, 8.94% and 11.62% higher than the baseline model on average. This work can recommend appropriate departments for patients, help hospitals effectively triage patients and improve patient experience.

In the future, we will further mine the characteristics of each outpatient department, because there are still many hidden patterns and rules to be discovered to achieve better classification results. Meanwhile, we also try to address performance issues with short text and apply Dual-CGA to other fields, such as sentiment analysis and disease prediction.
